# Acute coronary syndrome patients admitted to a cardiology vs non-cardiology service: variations in treatment & outcome

**DOI:** 10.1186/s12913-017-2294-0

**Published:** 2017-05-16

**Authors:** Deirdre E. O’Neill, Danielle A. Southern, Colleen M. Norris, Blair J. O’Neill, Helen J. Curran, Michelle M. Graham

**Affiliations:** 1 Division of Cardiology and Department of Medicine, and Mazankowski Alberta Heart Insitute, University of Alberta, Edmonton, Canada; 2 Department of Public Health Sciences, University of Calgary, Calgary, Canada; 3 Division of Cardiology and Department of Medicine, Dalhousie University, Halifax, Canada; 4 Division of Cardiology, University of Alberta, 2C2 WMC, 8440 112 St, Edmonton, AB T6G 2B7 Canada

**Keywords:** Acute coronary syndrome, Myocardial infarction, Specialist, Generalist, Non-cardiology

## Abstract

**Background:**

Specialized cardiology services have contributed to reduced mortality in acute coronary syndromes (ACS).  We sought to evaluate the outcomes of ACS patients admitted to non-cardiology services in Southern Alberta.

**Methods:**

Retrospective chart review performed on all troponin-positive patients in the Calgary Health Region identified those diagnosed with ACS by their attending team. Patients admitted to non-cardiology and cardiology services were compared, using linked data from the Alberta Provincial Project for Outcomes Assessment in Coronary Heart Disease (APPROACH) registry and the Strategic Clinical Network for Cardiovascular Health and Stroke.

**Results:**

From January 1, 2007 to December 31, 2008, 2105 ACS patients were identified, with 1636 (77.7%) admitted to cardiology and 469 (22.3%) to non-cardiology services. Patients admitted to non-cardiology services were older, had more comorbidities, and rarely received cardiology consultation (5.1%). Cardiac catheterization was underutilized (5.1% vs 86.4% in cardiology patients (*p* < 0.0001)), as was evidence-based pharmacotherapy (*p* < 0.0001). Following adjustment for baseline comorbidities, 30-day through 4-year mortality was significantly higher on non-cardiology vs. cardiology services (49.1% vs. 11.0% respectively at 4-years, *p* < 0.0001).

**Conclusion:**

In a large ACS population in the Calgary Health Region, 25% were admitted to non-cardiology services. These patients had worse outcomes, despite adjustment for baseline risk factor differences. Although many patients were appropriately admitted to non-cardiology services, the low use of investigations and secondary prevention medications may contribute to poorer patient outcome. Further research is required to identify process of care strategies to improve outcomes and lessen the burden of illness for patients and the health care system.

## Background

Coronary Care Units (CCU) have been shown to contribute to a reduction in mortality in acute coronary syndrome (ACS) since the 1960’s [1–9]. This is thought to be related to more intensive monitoring, timely recognition and treatment of life-threatening arrhythmias, and more frequent prescription of evidence-based medications [4, 5].

Many groups have investigated the effect of specialist care versus generalist care in a variety of medical conditions, including chronic obstructive pulmonary disease, HIV, heart failure and acute myocardial infarction [10–15]. As medical expenditures escalate, increasing attention has been paid to the cost differences in the treatment of ACS by cardiologists versus non-cardiologists and the impact this may have on prognosis [16, 17].

To date, the literature has been inconsistent on the influence of non-cardiology management of ACS. Shreiber et al. found ACS patients admitted to a cardiology service were more likely to be treated with evidence-based secondary prevention medications. They were also more likely to receive angiogram and angioplasty [18]. Similarly, Nash and colleagues demonstrated that ACS patients treated by a cardiologist had a 30% lower in-hospital mortality compared to those treated by a general practitioner or internist. [19] However, other studies have shown that the average patient admitted to a cardiology ward is much younger, with fewer comorbidities and an attenuated benefit with specialist treatment is seen when outcomes are adjusted for baseline risk factor differences [10, 20].

With the advancing age of the population, multiple non-cardiac comorbidities may be present in ACS patients, making it at times more appropriate to admit certain ACS patients to non-cardiology services. In addition, should a patient admitted with a different medical problem subsequently develop an ACS during hospitalization, transfer to cardiology may not be ideal.

Our objective was to evaluate the investigation and treatment of ACS patients admitted to a cardiology service versus a non-cardiology service, to determine whether admitting service was associated with the patient outcome of mortality or re-hospitalization. We also sought to evaluate any differences in the processes of care on these services.

## Methods

Calgary is a city in Alberta, Canada with a population of 1,149,552 (2013 Census). The Calgary Health Region has three major hospitals, of which two are regional centers and one is a tertiary care center with cardiac catheterization capabilities. All three hospitals in this region are teaching hospitals affiliated with the Faculty of Medicine at the University of Calgary.

Cardiac troponin is a protein complex that regulates contraction of cardiac muscle. The measurement of troponin is essential to the work-up of ACS and it is currently accepted that a cardiac troponin value exceeding the 99^th^ percentile for a reference control group, in the setting of clinical history suggestive of ACS, is consistent with myocardial necrosis [21]. Therefore over a period of January 1, 2007 to December 31, 2008, all inpatients with positive troponin values within the Calgary Health Region were identified. This time period was chosen as the value of troponin in the diagnosis of ACS was well-established and therefore it would be ordered on patients where the diagnosis of ACS was being considered. At the time of data collection, the Calgary Health Region was measuring cardiac troponin T (cTnT), using the Roche Elecsys 2010 Modular Analytics E170. Normal value for troponin T using this assay is <0.03 nm/L and reported values of 0.03-40.0 nm/L exist. This compilation of standardized laboratory data therefore provided us the opportunity to identify individuals from all hospitals in Calgary who have undergone cTnT testing. Outside of the Calgary Health Region, troponin measurements are performed using multiple assays and were not available in a central repository. Therefore, patients whose primary residence was outside the Calgary Health Region or those transferred from institutions outside the region were excluded from the analysis. Patients whose troponin elevations occurred around the time of elective PCI or as a result of cardiac surgery or an electrophysiology procedure were also excluded.

A retrospective chart review, completed by an individual with medical training and specific knowledge of cardiovascular disease, was performed on all troponin-positive patients, identifying those the admitting service documented ACS as the cause of the elevated troponin. Further data on diagnostic tests, medications prescribed and mortality was then collected using the Alberta Provincial Project for Outcomes Assessment in Coronary Heart Disease (APPROACH) registry as well as Discharge Admission Data, provided by the Cardiovascular Health & Stroke Strategic Clinical Network of Alberta Health Services. In addition, an experienced cardiologist, blinded to all patient data, interpreted all electrocardiograms (ECG) performed around the time of the troponin elevation. We compared the use of specific cardiac investigations and treatments of those patients admitted to a cardiology service, with those admitted to a non-cardiology service. Additionally, we compared mortality and re-hospitalization, to see whether outcome differences existed between these two groups.

### Data sources

APPROACH is a population based clinical registry, that captures all patients undergoing cardiac catheterization in Alberta, Canada (2013 Census Population 3,828,484) since 1995 and follows them longitudinally to determine patient outcome [22]. Within the APPROACH database there are several modules; including an admission module which tracks all patients (regardless of having undergone cardiac catheterization) admitted with ACS (STEMI, NSTEMI, UA) across Southern Alberta. The APPROACH registry also contains detailed information regarding patient demographics, cardiac risk factors, comorbidities, and results of cardiac catheterization and revascularization procedures. Comorbidities are subsequently verified through a data enhancement procedure to ensure comorbidity data are accurate and there are no missing data [23, 24]. Mortality data for all patients in the database is ascertained through semiannual linkage to the Alberta Bureau of Vital Statistics.

The Cardiovascular Health & Stroke Strategic Clinical Network is an Alberta-wide team of healthcare professionals, researchers and policy makers who are knowledgeable about cardiovascular health and work to improve its prevention, treatment and management, through accessing and supporting research. Their support provided access to diagnostic tests including echocardiograms, myocardial perfusion imaging and CT scans and also allowed linkage of administrative data for readmissions.

### Statistical analysis

Patients were grouped into those admitted to cardiology service, including those admitted to a cardiology ward or coronary care unit, and those admitted to a non-cardiology service, including all other services. The non-cardiology service patients were admitted to generalist or specialist physicians in a field other than cardiology. Baseline clinical characteristics were compared using chi-square tests. Kaplan Meier plots and log-rank tests were used to determine and compare crude mortality rates for these two groups. Mortality was then adjusted for age, sex and Charlson comorbidity index.

The Charlson comorbidity index is a weighted index that categorizes patients based upon comorbidities and the risk of mortality or resource use associated with such comorbidities. The sum of all of the weights results in an overall comorbidity score and is used to predict 10-year mortality. This index was first published in 1987 and has since been modified by various groups to include more comorbidity categories and age [25–27].

Analyses were performed using SAS, version 9.3 (Cary, NC).

## Results

From January 1, 2007 to December 31, 2008, 4,860 patients with a positive cardiac troponin T were admitted to hospital in the Calgary Health Region. Of these, the attending team diagnosed 2,105 (43.4%) as having occurred as a result of ACS. Of those deemed to have had an ACS, 1,636 (77.7%) were admitted to a cardiology service and 469 (22.3%) were admitted to a non-cardiology service.

Table 1 shows the baseline characteristics of both patient groups. Those admitted to non-cardiology services were significantly older (79.6 years vs. 62.9 years, *p* < 0.0001), with more comorbidities, with the exception of hypertension and current smoking.

**Table 1:** Baseline characteristics

Characteristic	Cardiology service *N* = 1636	Non-cardiology service *N* = 469	*P*-value
Mean Age (Std dev.)	62.9 (13.4)	79.6 (11.9)	<0.0001
Male sex	1178 (72.0)	240 (51.2%)	<0.0001
Comorbidities			
Cerebrovascular Disease	21 (1.3%)	51 (10.9%)	<0.0001
Diabetes	340 (10.8%)	151 (32.2%)	<0.0001
Hypertension	885 (54.1%)	218 (46.5%)	0.004
Hyperlipidemia	317 (19.4%)	14 (3.0%)	<0.0001
Peripheral vascular disease	25 (1.5%)	26 (5.5%)	<0.0001
Heart Failure	150 (9.2%)	176 (37.5%)	<0.0001
Prior MI	60 (3.7%)	23 (4.9%)	0.225
Pulmonary Disease	67 (4.1%)	100 (21.3%)	<0.0001
Malignancy	26 (1.6%)	63 (13.4%)	<0.0001
Liver/GI Disease	13 (0.8%)	25 (5.3%)	<0.0001
Renal Disease	83 (5.1%)	151 (32.2%)	<0.0001
Current Smoker	242 (14.8%)	22 (4.7%)	<0.0001
Dementia	25 (1.5%)	66 (14.1%)	<0.0001

*Std dev* standard deviation, *MI* myocardial infarction, *GI* gastrointestinal

Of those admitted to a non-cardiology service, family medicine, nephrology and internal medicine were the most common admitting services, at 45%, 21.3% and 17.7% respectively. Despite the documented diagnosis of ACS, very few cardiology consultations and transfers of care occurred, with only 4.5% of patients admitted to non-cardiology services receiving a cardiology consult and subsequent transfer to a cardiology service and only 0.6% receiving consultation alone (Table 2).

**Table 2:** Admitting service and consultation

Characteristics	Non-cardiology service *N* = 469
Admitting Physician Service	
Family Medicine	211 (45.0%)
Nephrology	100 (21.3%)
Internal Medicine	83 (17.7%)
Critical Care	47 (10.0%)
Neurology	18 (3.8%)
Surgery	10 (2.1%)
Cardiology Consult	
Consulted + transferred	21 (4.5%)
Consulted (no transfer)	3 (0.6%)

When an experienced cardiologist analyzed all ECGs of those admitted with ACS, 3.2% of those admitted to cardiology and 4.1% admitted to a non-cardiology service did not have an ECG performed (NS). Of the ECG changes identified, ST segment changes (elevation or depression) occurred significantly more often in those patients admitted to cardiology, whereas T wave abnormalities were most common in those admitted to non-cardiology services. Additionally, more patients admitted to non-cardiology services did not have a follow-up ECG performed (5.1% vs. 24.7%, *p* < 0.0001) (Table 3).

**Table 3:** Investigations & treatment

Investigations	Cardiology service *N* = 1636	Non-cardiology service *N* = 469	*P*-value
ECG Findings			
No ECG	53 (3.2%)	19 (4.1%)	0.455
ECG Changes			
ST elevation	685 (41.9%)	34 (7.3%)	<0.0001
ST depression	417 (25.5%)	77 (16.4%)	<0.0001
T wave changes	575 (35.2%)	151 (32.2%)	0.312
Bundle branch block	123 (7.5%)	55 (11.7%)	0.004
Dynamic ECG Changes	117 (7.2%)	117 (25.0%)	<0.0001
No follow-up ECG	84 (5.1%)	116 (24.7%)	<0.0001
Troponin Values			
Mean (Std. Dev.)	1.69 (3.53)	0.48 (1.01)	<0.0001
Minimum	0.10	0.10	
Maximum	39.40	11.89	
Tests Performed			
Angiogram during index admission	86.4%	5.1%	<0.0001
MIBI	4.5%	2.8%	0.103
Echocardiogram	2.4%	43.3%	<0.0001
CT Chest	0	8.5%	<0.0001
Medications			
Aspirin	91.9%	80.8%	<0.0001
Clopidogrel	83.3%	31.8%	<0.0001
Beta Blocker	84.4%	73.6%	<0.0001
Ace Inhibitor	74.9%	62.9%	<0.0001
Statin	79.1%	56.9%	<0.0001

*ECG* electrocardiogram, *ST* ST segment, *Std Dev* Standard deviation, *MIBI* technetium sestamibi myocardial imaging scan, *CT* computerized tomography

Cardiac troponin T values were on average higher in those admitted to cardiology (1.69 μm/l vs. 0.48 μm/l, *p* < 0.0001), as were maximum troponin values achieved (39.40 μm/l vs 11.89 μm/l, *p* < 0.0001). Those admitted to cardiology underwent angiogram significantly more often, with 86.4% undergoing angiogram on their index admission, compared to 5.1% in those admitted to non-cardiology services. Myocardial perfusion scans were performed infrequently on index admission in both the cardiology and non-cardiology groups. Echocardiogram and CT chest to rule out pulmonary embolism were more common in those patients admitted to non-cardiology services (2.4% vs. 43.3% and 0 vs. 8.5%, respectively, both *p* values <0.0001), as seen in Table 3.

Through retrospective chart review, the use of evidence-based medications for secondary prevention was identified. Aspirin, clopidogrel, beta blockers, ACE inhibitors and statin drugs were all used significantly less often in those admitted to a non-cardiology service when compared to those admitted to cardiology (Table 3).

**Fig. 1:**
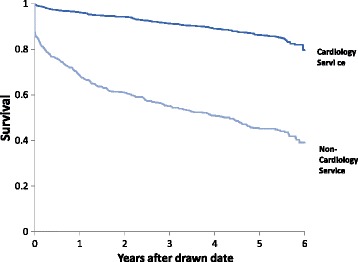
Kaplan Meier mortality curve

As shown in Table 4, the 30-day mortality was significantly lower for patients admitted to cardiology, compared to those admitted to a non-cardiology service (2.6% vs. 20.3%, respectively, *p* < 0.0001). Mortality through to 4-years remained higher in those admitted to a non-cardiology service (11% vs 49.1%, respectively, *p* < 0.0001). This is also represented in the Kaplan Meier survival curve presented in Fig. 1. In addition, 1-year readmission rates were also significantly higher for those admitted to a non-cardiology service (26.7% vs. 39.4%, *p* < 0.0001).

**Table 4:** Mortality

Outcome	Cardiology service *N* = 1636	Non-cardiology service *N* = 469	*P*-value	Crude OR (95% CI)	Adjusted^a^ OR (95% CI)
30-day mortality	43 (2.6%)	95 (20.3%)	<0.0001	4.19 (3.19,5.49)	3.33 (2.42,4.59)
1-yr mortality	62 (3.9%)	140 (31.5%)	<0.0001	3.39 (2.89,3.99)	2.25 (1.86,2.72)
2-yr mortality	92 (5.7%)	173 (39.0%)	<0.0001	3.25 (2.82,3.74)	2.06 (1.75,244)
3-yr mortality	140 (8.7%)	200 (45.1%)	<0.0001	2.93 (2.58,3.33)	1.83 (1.58,2.13)
4-yr mortality	177 (11.0%)	218 (49.1%)	<0.0001	2.80 (2.48,3.16)	1.70 (1.47,1.96)
30-day readmission	150 (9.3%)	59 (13.3%)	0.014		
1-yr readmission	430 (26.7%)	175 (39.4%)	<0.0001		

*Yr* year

^a^Adjusted for age, sex and Charlson comorbidity index

Reference group = cardiology ward

When outcomes are adjusted for age, sex and Charlson comorbidity index, 30-day through to 4-year point estimates for mortality remain significant for those admitted to a non-cardiology service (Table 4). The odds ratio for mortality at 30-days in the non-cardiology group is 3.33 (95% CI 2.42-4.59) and is still significant at 1.70 (95% CI 1.47-1.96) at 4-years when compared to the cardiology group.

## Discussion

We have found that in a modern major metropolitan region, about 25% of patients diagnosed with ACS were admitted to non-cardiology services. In keeping with previous literature, these patients were on average older and had more comorbidities. Our findings support the “specialist effect” for patient outcome, as even after adjustment for age, sex and baseline risk factor differences, we found short and longer term mortality to be significantly higher for those admitted to a non-cardiology service, as compared to those admitted to cardiology.

Previous literature has shown superior treatment and outcome for ACS patients treated by cardiologists as compared to non-cardiology-trained physicians [18, 19]. However, the ACS patient population admitted to cardiology is often younger, with fewer comorbidities and can result in an attenuated mortality benefit [10, 20]. In accordance with the literature, our study showed patients admitted to cardiology were starkly different from those admitted to non-cardiology services. Patients on non-cardiology services were older with more numerous comorbidities, including cerebrovascular disease, diabetes, pulmonary disease, malignancy and renal disease. However, even with adjustment, mortality was significantly higher in patients treated by non-cardiology services, suggesting outcome is also associated with admitting services.

Dynamic ECG changes were common in those admitted to non-cardiology services, occurring in 25% of patients. While occurring significantly less frequently than in the cardiology group, ST elevation still occurred in over 7% of the patients and ST depression occurred in 16%. However, only 5% underwent angiogram during their index admission, and less than 3% underwent non-invasive risk stratification with myocardial perfusion imaging. This may be the result of the infrequent cardiology consultation noted in this group, as in the Calgary Health Region, a cardiology consultation is required in order to have an angiogram performed. Follow-up ECGs were also completed less frequently, with 24.7% having no follow-up ECG performed on a non-cardiology service, as compared to only 5.1% in the cardiology group (<0.0001). This allows the possibility for further signs of ischemia to have been missed in the period of time following the recognized troponin elevation.

Another process of care difference was identified in the use of echocardiogram. Those on non-cardiology services underwent echocardiogram more frequently than those admitted to cardiology. In the Calgary health region, at the time of data collection, standard practice was to perform a left ventriculogram and quantitative ejection fraction calculation at the time of angiogram, rather than echocardiogram, which was usually left to outpatient follow up visits. This likely accounts for the discrepant use of this test.

The differences outlined clearly demonstrate divergent processes of care used for patients admitted to cardiology versus non-cardiology services. It is possible that patients admitted to non-cardiology services were not candidates for invasive investigation with cardiac catheterization. However, secondary prevention medications have few adverse effects compared to their benefits and were still significantly underprescribed. Multiple studies support the use of beta-blockers, statins and ASA as secondary prevention medications in the elderly and even in the very elderly (>80 years) population [28–33]. Therefore current evidence would support considering the use of these medications on an individual basis where appropriate.

The decision to treat patients with ACS on a non-cardiology service may be appropriate in many cases, due to comorbidities and/or other acute diagnoses that benefit from the expertise of generalists or noncardiology subspecialists. However, the investigation and management of these patients could potentially be improved with more formal or defined collaborative consultation with cardiology. Willison et al. found that patients with acute myocardial infarction treated by a generalist with cardiology consultation had similar treatment to those treated by a cardiologist alone [34]. Improved patient outcome with consultation has also been shown in other medical specialties, with several studies finding improved mortality associated with infectious disease consultation [35–37]. Therefore, as Willison et al. concluded, in the generalist/specialist care debate, the focus should be on improving consultation between generalists and specialists to improve quality of care [34].

We believe that we have identified an important process of care issue for ACS patients admitted to non-cardiology services. Standardized order sets and decision-support tools present physicians with a template for ordering diagnostic tests, medications and other items, based upon current evidence. Standardized order sets have been shown to improve quality of care, adherence to guideline-based therapies and in many cases, decrease mortality [38–42]. More specifically, Ellerbeck et al. found the use of standardized order sets significantly increased the use of aspirin and beta blockers on admission and discharge of ACS patients and Milani et al. found computerized decision support increased the likelihood of achieving so-called “perfect care” in ACS and was an independent predictor of survival [42, 43]. With the variation in outcome based upon admitting service seen in our study, the introduction of such a processes throughout all areas of the hospital, triggering, for example, a cardiology consult or the use of certain evidence-based medications, could result in more standardized treatment of all ACS patients, regardless of care location. A quality improvement study comparing the outcome of patients with ACS before and after introduction of a hospital-wide ACS standardized order set could be helpful in promoting uptake of such a process of care change in the future.

This study does have limitations. It is an observational study and therefore residual confounding can occur. In particular, goals of care were not routinely collected; therefore it is possible that patients were not investigated because of pre-stated goals of care requesting to forgo aggressive medical management. However, this is not likely the majority of our cohort, given survival beyond hospitalization. In addition, troponins were not collected in an unselected manner, but were rather ordered by the attending service, when considered indicated. Certainly, it is possible that other ACS patients may have remained undiagnosed due to lack of troponin measurement. Additionally, we determined the diagnosis of ACS via retrospective chart review. It is possible that the some of the patients in our study may have actually experienced a type II MI, or another cause of troponin elevation. However, rightly or wrongly, the admitting team felt that the diagnosis was ACS, and their process of care was evaluated accordingly. Lastly, it is difficult to completely elucidate why a patient was admitted to a non-cardiology service or why cardiology consultation occurred so infrequently via chart review as this was not well documented. However, with such few cardiology consultations having occurred, it is clear that this decision is often being made without the input of cardiology.

## Conclusion

In conclusion, one quarter of ACS patients admitted to tertiary care teaching hospitals in a major metropolitan city, were admitted to non-cardiology services. These patients were on average older and had more comorbidities. Despite a diagnosis of ACS, cardiology consultation occured rarely, and significantly fewer evidence-based secondary prevention medications were prescribed. Significantly worse outcomes were identified for these patients, even after adjustment for age, sex and comorbidity. Although many of these patients may be appropriately treated on services other than cardiology, the low use of investigations and secondary prevention medications may be contributing to the poorer outcomes. Therefore, additional work is necessary to standardize the investigation and treatment of ACS and to encourage collaboration between specialties in order to improve outcomes and lessen the burden of illness for patients and the health care system.

## Abbreviations

ACE: Angiotensin converting enzyme
ACS: Acute coronary syndromel
APPROACH: Alberta Provincial Project for Outcomes Assessment in Coronary Heart Disease
CCU: Coronary care units
CT: Computerized tomography
cTnT: Cardiac troponin T
ECG: Electrocardiogram
NSTEMI: Non-ST elevation myocardial infarction
STEMI: ST elevation myocardial infarction
UA: Unstable angina
